# Mr. Docker, on Opiate Friction

**Published:** 1800-02

**Authors:** Tho. Docker

**Affiliations:** Surgeon to the Forces. Deal


### *To the Editors of the Medical and Phyſical Journal.*

GENTLEMEN,

THE ſucceſs which attended the uſe of the opiate friction in ſeveral caſes related by Mr. Ward, of Mancheſter, induced me to try it in the following, wherein it was attended with the moſt beneficial effects. If you think it worthy a place in your valuable Journal, you are at liberty to inſert it.

I remain, GENTLEMEN,
Your very humble ſervant,
THO. DOCKER,
Surgeon to the Forces.

*Deal, Dec.* 22, 1799.

JOHN BAYLIS, of the Third Regiment of Guards, was received into the Military Hoſpital at this place, from Holland, as a venereal patient. Upon examination, I found a conſiderable ſphacelation in the groin, from a bubo which had been opened

ſom fome time. It was then rapidly increafing, and from the general ftate of his health, I had every reafon to fear a fatal termination. The part was ordered to be fomented, and the carrot poultice applied; he had cordials with bark and opium prefcribed, and as much wine as he could take; but notwithftanding, the fphacelation ftill continued to advance. Oct. 12. This morning I found him extremely ill, the attendants thought he would have died in the night. His ftomach rejected every thing he took; his pulfe could fcarcely be counted; a diarrhœa had come on, and his groin prefented a moft fhocking appearance. I concluded that a few hours would have terminated his exiftence. It now occurred to me, that opium, introduced by friction, would, probably, be attended with advantage, and I determined to try it as the laft refource. I accordingly directed the following to be immediately rubbed into the thighs and legs.

Tinct. opii q. ʒij. Camph. ℥fs.

Ol. oliv. ℥ij. vitel. ovi q. s. M.

I vifited him a fhort time afterwards, and found him, I thought, more compofed and lefs languid, and he had taken fome wine without ficknefs. I directed the fame quantity to be ufed again, and to be repeated in the courfe of the night, with the addition of one drachm of tinct. opii. Oct. 13. This morning evidently better, had paffed the night with fome fleep, and the inflammation about the edges of the ulcer confiderably abated; the diarrhœa had alfo ftopped. I directed three drachms of the tinct. opii to be ufed three times in the courfe of the day. I faw him in the evening; he had taken a good deal of wine and fome foup without any ficknefs, and his pulfe was better; four drachms of tinct. opii were ordered to be ufed during the night. 14th. This morning he appeared in a progreffive ftate of recovery, though he had flept but little, and perfpired profufely; but he had had a regular ftool, and the inflammation about the edges of the ulcer had ftopped, and its furface looked lefs gangrenous. I directed four drachms to be rubbed in every five hours. 15th. A good deal of compofed fleep during the night; the inflammation and fpreading intirely ftopped; the flough feparated from the found parts, and the ulcer now looked quite clean. He has taken a little wine during the laft twenty-four hours, and eat fome fifh with appetite; the friction continued. 16th. Slept the greateft part of the night, and awoke greatly refrefhed; friction continued. 17th, The carrot poultice omitted, and the ulcer treated with fimple dreffings; the friction continued, and he was directed to take fome infufion of bark. 19th. As the bark had created naufea, I ordered it to be omitted; and as his health and appetite now rapidly improves, I diminifhed the opium one third. 24th. He has now a very good good appetite, and the ulcer heals rapidly; the opium diminished one half. 27th. The opiate friction intirely left of, and he is directed to take the decoction of bark, with elixir of vitriol. Nov. 13th. The ulcer is now almost healed, and he is, in other respects, in perfect good health.

